# Supplementary material for: PRC1-mediated epigenetic programming is required to generate the ovarian reserve
Source: Nat Commun. 2022 Aug 10;13:4510. doi: 10.1038/s41467-022-31759-6 (PMC9365831; doi:10.1038/s41467-022-31759-6)
Supplement: Supplementary file 1 — Supplementary Information [file 41467_2022_31759_MOESM1_ESM.pdf]

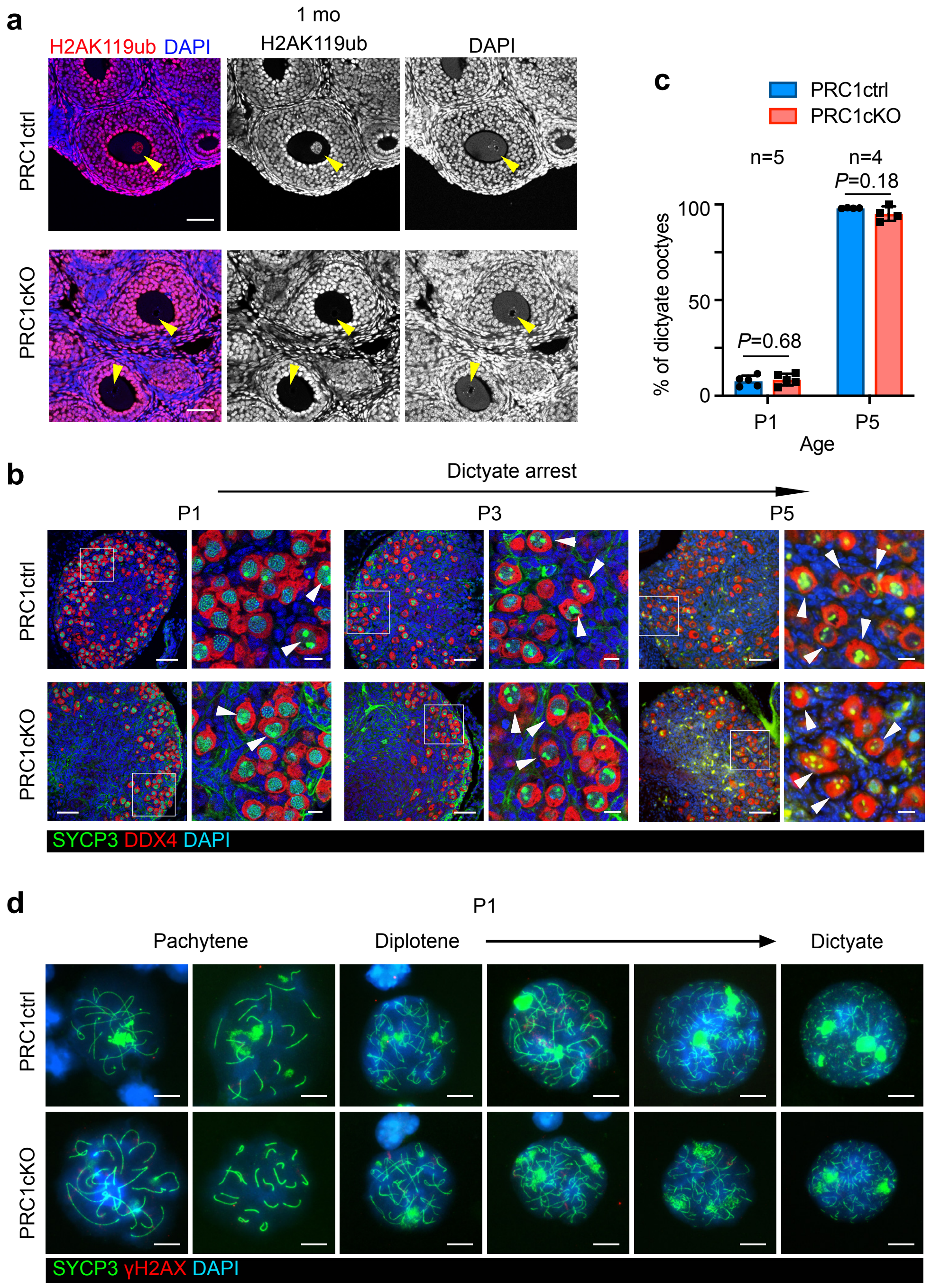

Supplementary Fig. 1: *Ddx4-Cre*-mediated PRC1 inactivation did not affect overall meiosis progression. a, Immunostaining of H2AK119ub in the ovaries of PRC1cKO and a control littermate at 1 month-of-age. Yellow arrowheads are showing the oocyte nucleus. Bars: 50 µm. At least three mice were analyzed for each genotype, and representative images are shown. **b,** Immunostaining of SYCP3 and DDX4 in the ovaries of PRC1ctrl and PRC1cKO at P1, P3, and P5. White arrowheads indicate the dictyate oocytes. Bars: 50 µm (10 µm in the boxed area). At least three mice were analyzed for each genotype, and representative images are shown. **c,** Corresponding quantifications of dictyate oocytes percentage are shown. n indicates the number of independent replicates used for analysis. Data are presented as mean values ± SD. Two-tailed unpaired t-tests. **d,** Chromosome spreads of P1 oocytes in PRC1ctrl and PRC1cKO immunostained for SYCP3 (green) and γH2AX (red). Bars: 10 µm. At least three mice were analyzed for each genotype, and representative images are shown. Source data are provided as a Source Data file.

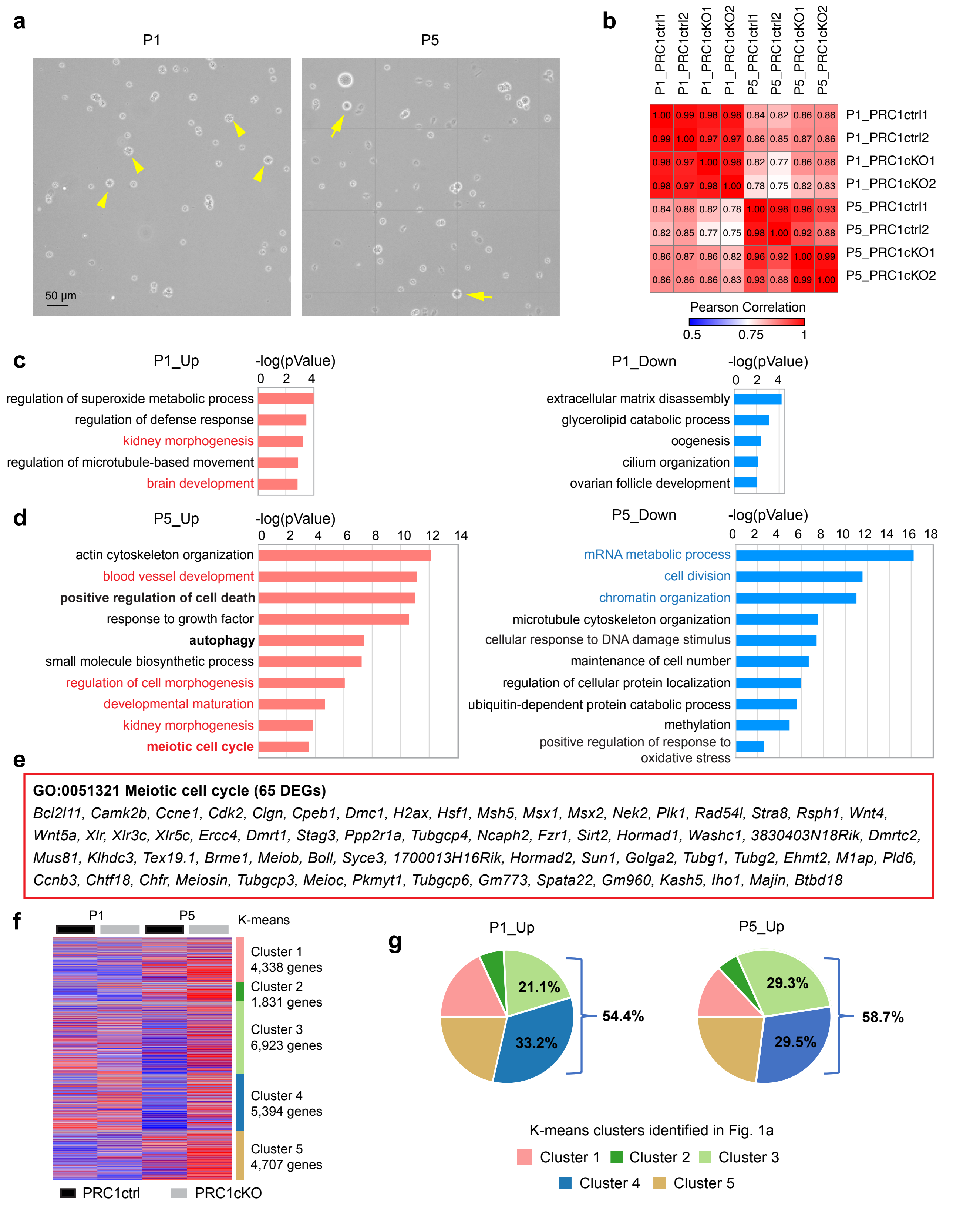

Supplementary Fig. 2: RNA-seq analysis of PRC1ctrl and PRC1cKO oocytes at P1 and P5. **a**, Representative images of ovarian cells after tissue dissociation at P1 and P5. Yellow arrowheads and arrows point to typical single non-growing oocytes manually picked up for RNA-seq analysis at P1 and P5, respectively. At least three independent biological replicates were analyzed. **b**, Heatmap showing the reproducibility between biological replicates in RNA-seq dataset. Pearson correlation values are computed by deeptools using two-sided Pearson method. **c, d,** Gene ontology analyses of the DEGs. Key GO terms of the DEGs are shown. P values were generated by Metascape using two-sided hypergeometric test. **e,** List of 65 DEGs in GO term "meiotic cell cycle." **f**, Heatmap showing gene expression in PRC1ctrl and PRC1cKO oocytes at P1 and P5 categorized into five k-means clusters (detected in wild-type POT in Fig. 1a). **g**. Pie charts showing cluster distributions (Cluster 1-5 identified in Fig. 1a) in up-regulated genes in PRC1cKO oocytes at P1 and P5. Source data are provided as a Source Data file.

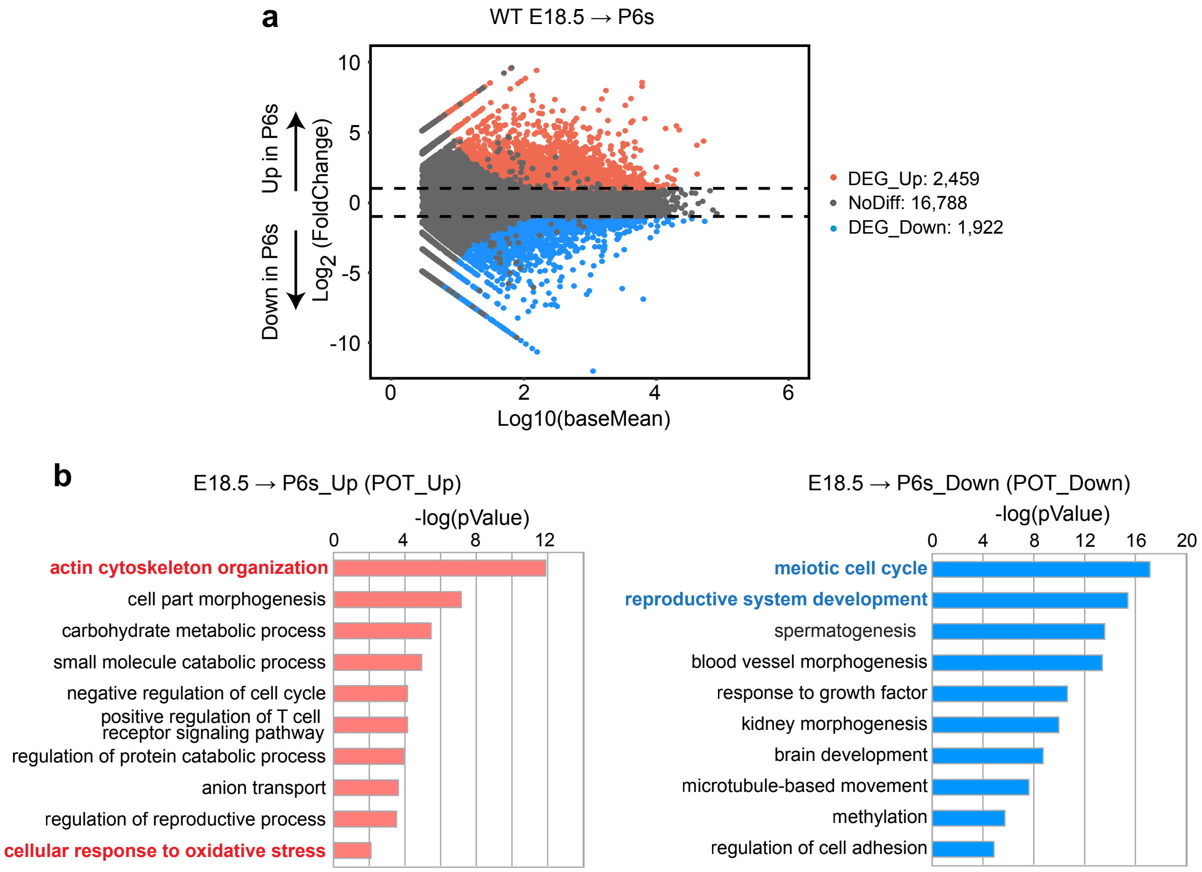

Supplementary Fig. 3: Identification of POT genes. **a**, Comparison of transcriptomes between wild-type E18.5 and P6 small (P6s) oocytes. DEGs (Log2FoldChange > 1, FDR < 0.05) are defined as POT genes and colored (red: up-regulated in wild-type P6s oocytes; blue: down-regulated in wild-type P6s oocytes), and numbers are shown. **b**, Gene ontology analyses of the POT genes. Top GO terms are shown. P values were generated by Metascape using two-sided hypergeometric test. Source data are provided as a Source Data file.

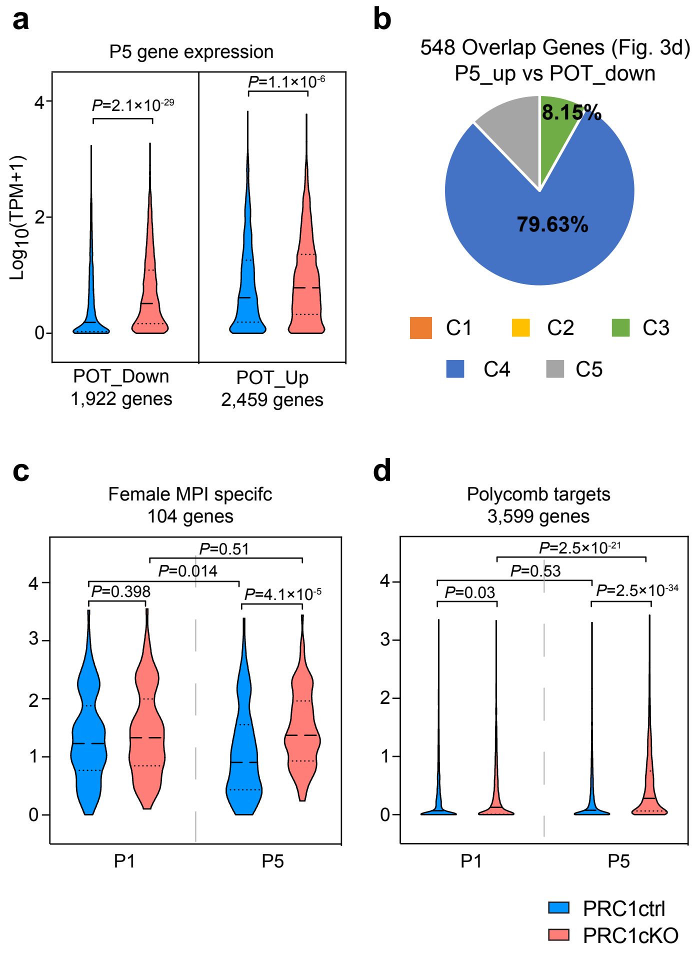

Supplementary Fig. 4: Gene expression in PRC1ctrl and PRC1cKO oocytes at P1 and P5. **a**, Violin plots showing expression level of POT gene groups in PRC1ctrl and PRC1cKO oocytes at P5. Two-tailed unpaired t-tests. **b**, A pie chart showing cluster distributions (Cluster 1-5 identified in Fig. 1a) in 548 overlapped genes (identified in Fig. 3d) between up-regulated DEGs in PRC1cKO oocytes at P5 and down-regulated POT genes in wildtype. **c**, **d**, Violin plots showing the expression level of gene groups as indicated in PRC1ctrl and PRC1cKO oocytes at P1 and P5, respectively. Central bars represent medians, the dotted lines represent 50% of the data points. Two-tailed unpaired t-tests. Source data are provided as a Source Data file.

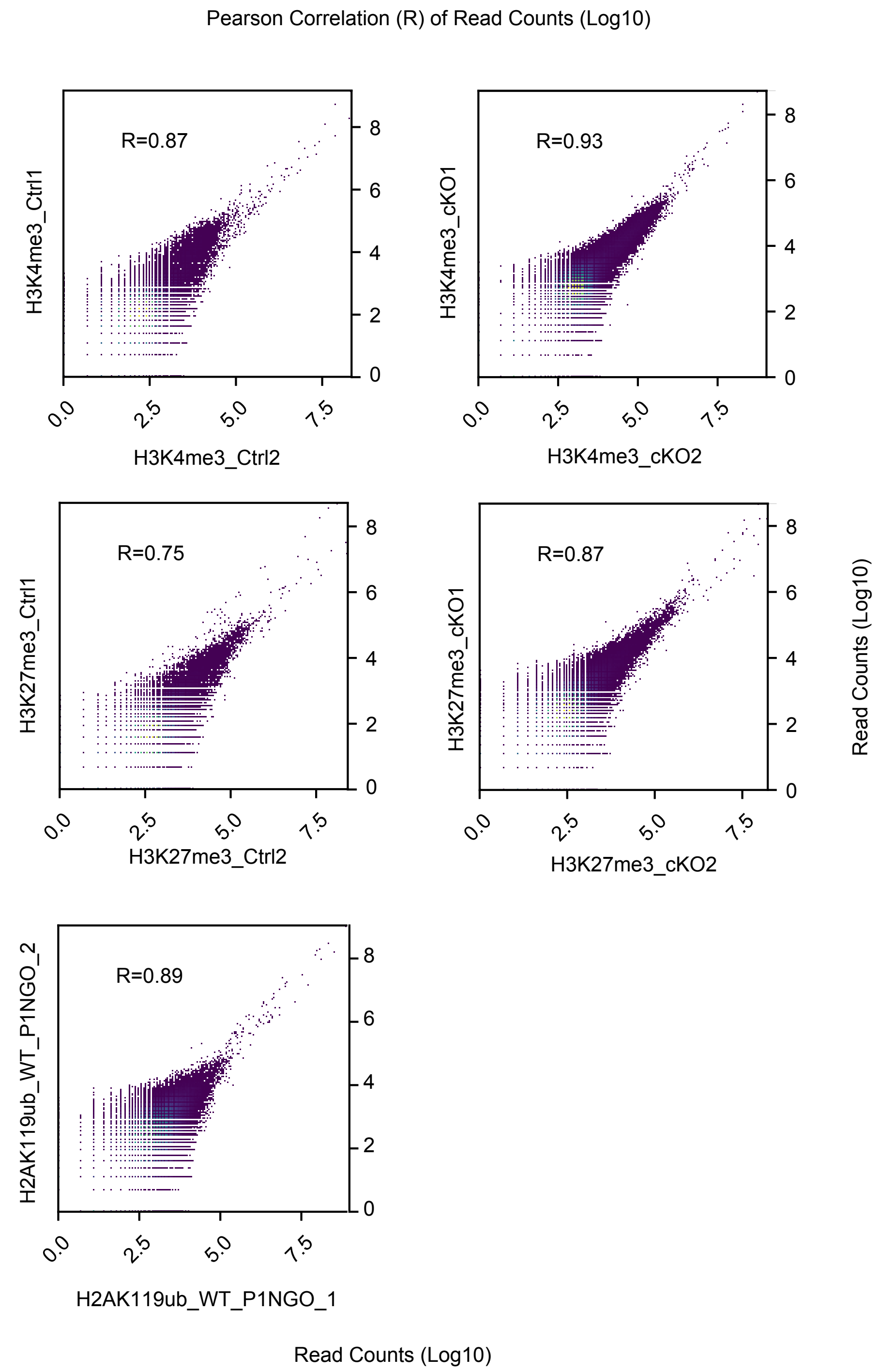

Supplementary Fig. 5: Biological replicates for CUT&RUN data. Scatter plots show the reproducibility between biological replicates in CUT&RUN data. Pearson correlation values (R) are shown.

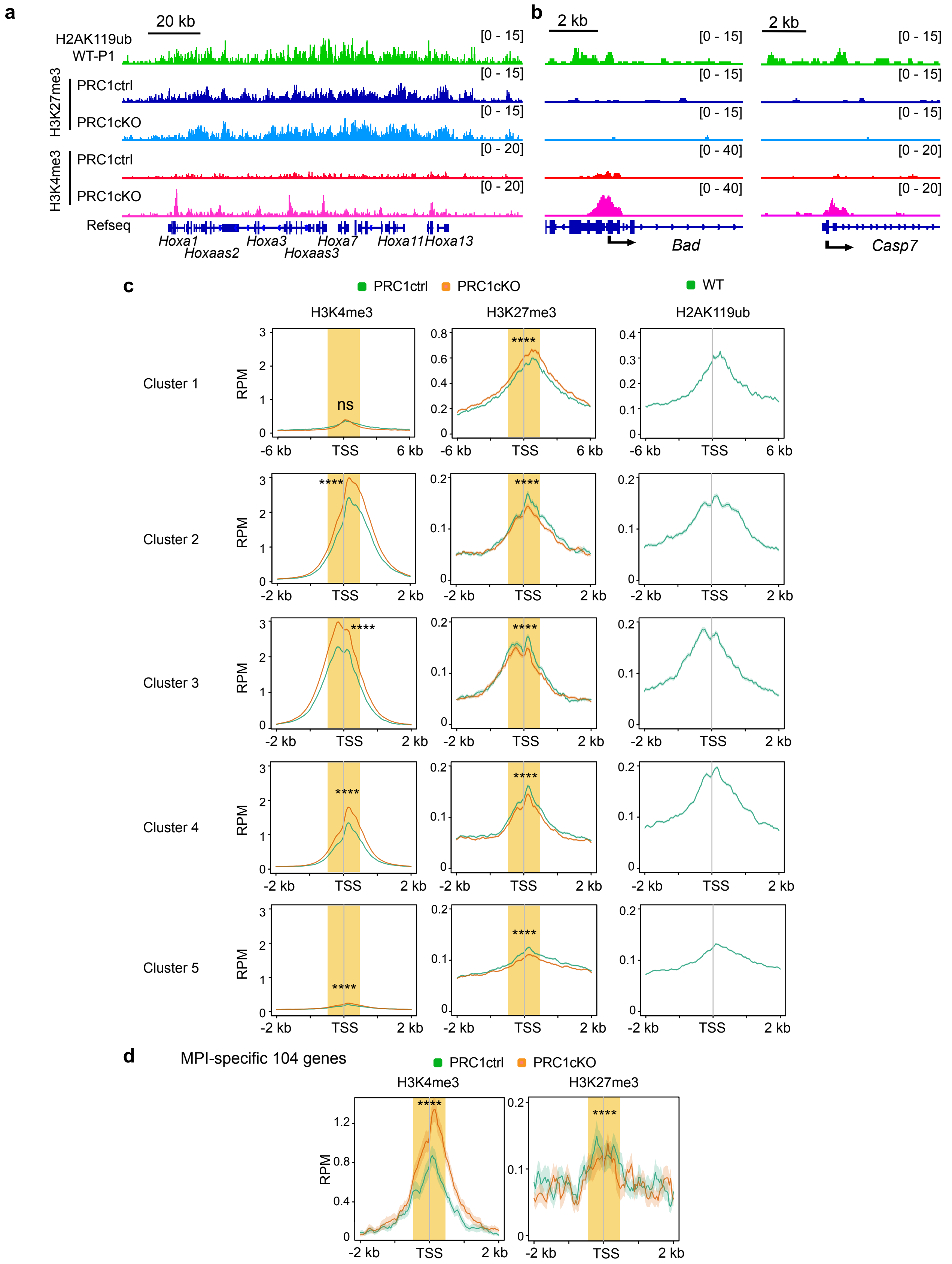

Supplementary Fig. 6: Removal of PRC1 affects the deposition of H3K4me3 and H3K27me3 in oocytes. **a**, **b**, Track views of *Hoxa* gene locus (**a**) and *Bad* and *Casp7* gene loci (**b**) showing H2AK119ub, H3K4me3, and H3K27me3 CUT&RUN peaks in P1 oocytes of indicated genotypes. Data ranges are shown in brackets. **c**, Average tag density plots showing H3K4me3, H3K27me3, and H2AK119ub enrichment at promoter regions for each k-means cluster (identified in Fig. 4c). **d**. Average tag density plots showing H3K4me3 and H3K27me3 enrichment at promoter regions for 104 MPI-specific genes. Wilcoxon rank sum test (two-tailed) was performed for read counts in the highlighted area (TSS ± 1500bp for Cluster 1 and TSS ± 500bp for the others). ns, not significant, $P = 0.11$; **** $P < 1.3 \times 10^{-5}$. Source data are provided as a Source Data file.

a

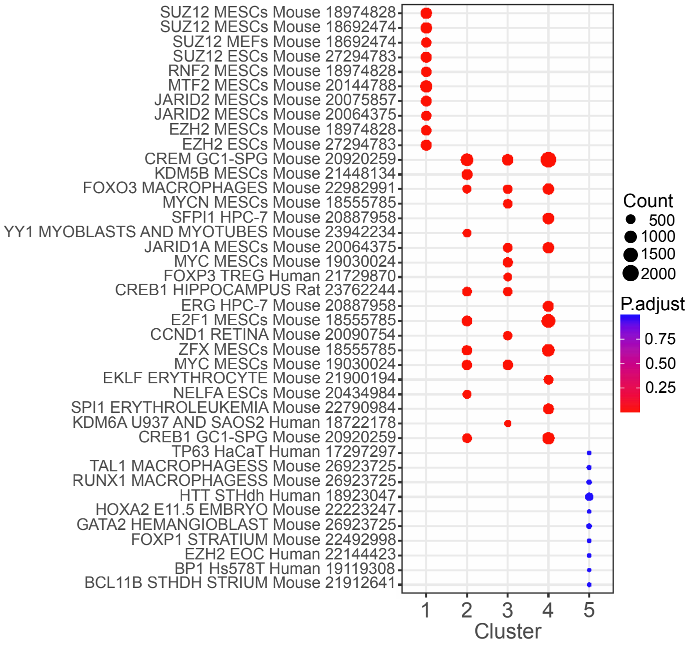

b

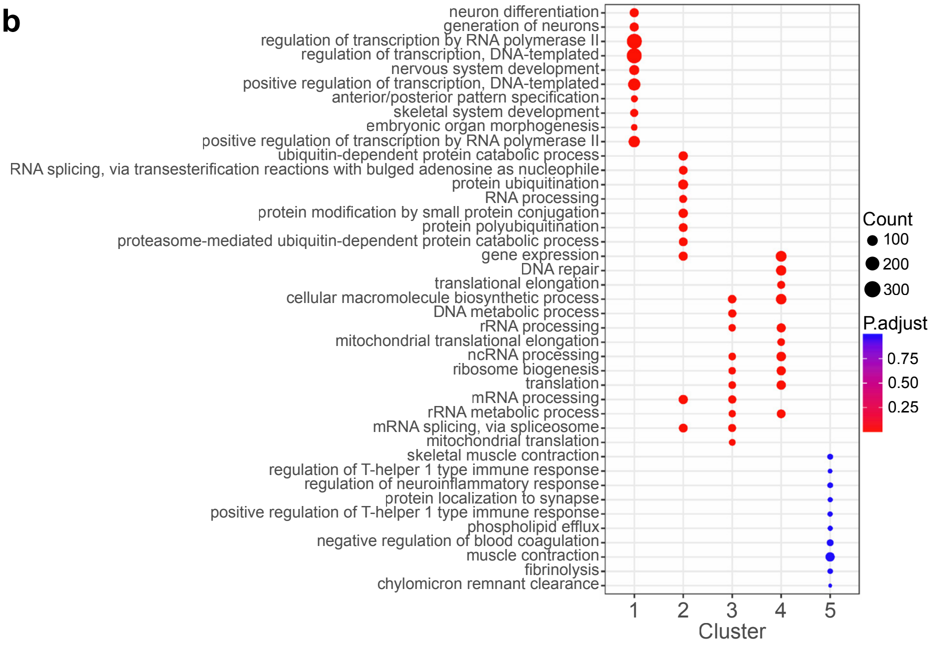

Supplementary Fig. 7: H2AK119ub, H3K4me3, and H3K27me3 target genes in P1 oocytes. a, **b**, Dot plots of ChIP-x Enrichment Analysis (ChEA) (**a**) and Gene Ontology Biological Process (GO-BP) term enrichment (**b**). The Top 10 enriched GO-BP terms and predicted transcription factors in the gene set of each k-means cluster (identified in Fig. 4c). ChEA enrichment shows the transcription factors and the cell and animal types used in the profiling experiments. Colors indicate the adjusted p-values using the Benjamini-Hochberg method for correction for multiple hypotheses testing, and dots size is proportional to gene count the number of genes enriched in the gene set library. Source data are provided as a Source Data file.

Supplementary Table 1. List of antibodies used in this study

Reagent or Resource	Source	Identifier
Antibodies		
DDX4	Abcam	CAT# ab13840; RRID:AB_443012
SYCP3	Novus	CAT# NB300-232; RRID:AB_2087193
SYCP3	Abcam	CAT# ab97672; RRID:AB_10678841
γH2AX	Millipore	CAT# 05-636; RRID:AB_309864
SYCP3 conjugated with Alexa 488 fluorophore	Abcam	CAT# ab205846; RRID:AB_10678841
γH2AX conjugated to Alexa 647 fluorophore	Millipore	CAT# 05-636-AF647; RRID:AB_309864
H2AK119ub	Cell Signaling Technology	CAT# 8240; RRID:AB_10891618
H3K27me3	Cell Signaling Technology	CAT# 9733; RRID:AB_2616029
H3K4me3	Cell Signaling Technology	CAT# 9751; RRID:AB_2616028
