# Supplementary material for: PRC1-mediated epigenetic programming is required to generate the ovarian reserve
Source: Nat Commun. 2022 Aug 10;13:4510. doi: 10.1038/s41467-022-31759-6 (PMC9365831; doi:10.1038/s41467-022-31759-6)
Supplement: Supplementary file 3 — Description of Additional Supplementary Files [file 41467_2022_31759_MOESM3_ESM.pdf]

**Description of Additional Supplementary Files**

File Name: Supplementary Data 1
Description: Gene IDs in five distinct gene-expression clusters revealed by clustering analysis of all expressed genes in wild-type oocytes

File Name: Supplementary Data 2
Description: Differentially expressed genes in P1 oocytes from PRC1cKO mice

File Name: Supplementary Data 3
Description: Differentially expressed genes in P5 oocytes from PRC1cKO mice

File Name: Supplementary Data 4
Description: Differentially expressed genes in wild-type oocytes during POT
