# Supplementary material for: PRC1-mediated epigenetic programming is required to generate the ovarian reserve
Source: Nat Commun. 2022 Aug 10;13:4510. doi: 10.1038/s41467-022-31759-6 (PMC9365831; doi:10.1038/s41467-022-31759-6)
Supplement: Supplementary file 8 — Reporting Summary [file 41467_2022_31759_MOESM8_ESM.pdf]

nature portfolio

Corresponding author(s): Satoshi H Namekawa

Last updated by author(s): Jun 24, 2022

Reporting Summary

Nature Portfolio wishes to improve the reproducibility of the work that we publish. This form provides structure for consistency and transparency in reporting. For further information on Nature Portfolio policies, see our Editorial Policies and the Editorial Policy Checklist.

Statistics

For all statistical analyses, confirm that the following items are present in the figure legend, table legend, main text, or Methods section.

n/a	Confirmed	
☐	☒	The exact sample size (*n*) for each experimental group/condition, given as a discrete number and unit of measurement
☐	☒	A statement on whether measurements were taken from distinct samples or whether the same sample was measured repeatedly
☐	☒	The statistical test(s) used AND whether they are one- or two-sided<br>*Only common tests should be described solely by name; describe more complex techniques in the Methods section.*
☒	☐	A description of all covariates tested
☒	☐	A description of any assumptions or corrections, such as tests of normality and adjustment for multiple comparisons
☐	☒	A full description of the statistical parameters including central tendency (e.g. means) or other basic estimates (e.g. regression coefficient) AND variation (e.g. standard deviation) or associated estimates of uncertainty (e.g. confidence intervals)
☐	☒	For null hypothesis testing, the test statistic (e.g. *F*, *t*, *r*) with confidence intervals, effect sizes, degrees of freedom and *P* value noted<br>*Give P values as exact values whenever suitable.*
☒	☐	For Bayesian analysis, information on the choice of priors and Markov chain Monte Carlo settings
☒	☐	For hierarchical and complex designs, identification of the appropriate level for tests and full reporting of outcomes
☐	☒	Estimates of effect sizes (e.g. Cohen's *d*, Pearson's *r*), indicating how they were calculated

Our web collection on statistics for biologists contains articles on many of the points above.

Software and code

Policy information about availability of computer code

Data collection	Microscopy images were acquired using Nikon confocal laser scanning microscope A1R. Sequencing data were collected using Illumina HiSeq 4000 and Novaseq 6000 platforms. No custom software was used in this study.
Data analysis	Trim-galore (version 0.6.6), HISAT2 (version 2.2.1), samtools (version 1.9), HTSeq package(version 1.6.0), DESeq2(version 1.28.1), RSEM(version 1.3.3), Metascape (http://metascape.org), Morpheus (https://software.broadinstitute.org/morpheus/), Bowtie2 (version 2.4.2), Picard tools (version 2.23.8), deepTools (version 3.5.0), bedtools (version 2.29.2), SEACR (https://seacr.fredhutch.org/), ngs.plot (version 2.63), Bamscale package(version 0.0.5), Enricher website (https://maayanlab.cloud/Enrichr/), ggplot2 (3.3.6). No custom code was used in this study.

For manuscripts utilizing custom algorithms or software that are central to the research but not yet described in published literature, software must be made available to editors and reviewers. We strongly encourage code deposition in a community repository (e.g. GitHub). See the Nature Portfolio guidelines for submitting code & software for further information.

Data

Policy information about availability of data

All manuscripts must include a data availability statement. This statement should provide the following information, where applicable:

- Accession codes, unique identifiers, or web links for publicly available datasets
- A description of any restrictions on data availability
- For clinical datasets or third party data, please ensure that the statement adheres to our policy

RNA-seq data and CUT&RUN data reported in this study were deposited to the Gene Expression Omnibus (accession no. GSE184208). Source data are provided with this paper.

March 2021

Field-specific reporting

Please select the one below that is the best fit for your research. If you are not sure, read the appropriate sections before making your selection.

☒ Life sciences ☐ Behavioural & social sciences ☐ Ecological, evolutionary & environmental sciences

For a reference copy of the document with all sections, see nature.com/documents/nr-reporting-summary-flat.pdf

Life sciences study design

All studies must disclose on these points even when the disclosure is negative.

Sample size	Sample size was chosen to ensure reproducibility of the results at affordable costs while ensuring significance in statistical tests. Sample sizes are indicated for all experiments. At least 2 and typically 3 or more independent experiments were carried out for most of the assays.<br>Bulk RNA-seq and CUT&RUN were obtained from biological duplicates.
Data exclusions	No data were excluded from analyses.
Replication	We confirmed consistent results between three independent biological replicates for all experiments. Key CUT&RUN and RNA-seq were performed in duplicates. The consistency between replicates were examined by pearson correlation and all datasets were included for analyses.
Randomization	For comparison between WT and mutants, randomization was not feasible as genotypes of mice needed to be first determined.
Blinding	Blinding was not considered as the authors need to be aware of genotypes/groups to perform analyses. No blinding should not affect interpretation as all our experiment measures were objective.

Reporting for specific materials, systems and methods

We require information from authors about some types of materials, experimental systems and methods used in many studies. Here, indicate whether each material, system or method listed is relevant to your study. If you are not sure if a list item applies to your research, read the appropriate section before selecting a response.

Materials & experimental systems

n/a	Involved in the study	
☐	☒	Antibodies
☒	☐	Eukaryotic cell lines
☒	☐	Palaeontology and archaeology
☐	☒	Animals and other organisms
☒	☐	Human research participants
☒	☐	Clinical data
☒	☐	Dual use research of concern

Methods

n/a	Involved in the study	
☐	☒	ChIP-seq
☒	☐	Flow cytometry
☒	☐	MRI-based neuroimaging

Antibodies

Antibodies used	Immunostaining of ovarian sections:<br>Sections were incubated with primary antibodies (anti-H2AK119ub: Cell Signaling Technology, 8240; anti-DDX4: Abcam, 13840; and anti-SYCP3: Novus, NB300-232 at 1:200 dilution).<br>Immunofluorescence of meiotic chromosome spreads:<br>Primary antibodies were used at the following dilutions: rabbit anti-SYCP3 (Novus, NB300-232), 1/500; mouse anti-γH2AX (Millipore, 05-636), 1/5000; mouse anti-SYCP3 (Abcam, ab97672), 1/5000; mouse anti-SYCP3 conjugated with Alexa 488 fluorophore (Abcam, ab205846), 1/500; mouse anti-γH2AX conjugated to Alexa 647 fluorophore (Millipore, 05-636-AF647), 1/500.<br>CUT&RUN library generation and sequencing:<br>The antibodies used were rabbit anti-H2AK119ub1 (1/100; Cell Signaling Technology; 8240), rabbit anti-H3K4me3 (1/100; Cell Signaling Technology; 9751), and rabbit anti-H3K27me3 antibody (1/100; Cell Signaling Technology; 9733).<br>Secondary antibodies:<br>Donkey Anti-Mouse IgG (H+L) Alexa Fluor 488, A-21202; Donkey Anti-Rabbit IgG (H+L) Alexa Fluor 555, A-31572; Donkey Anti-Rabbit IgG (H+L) Alexa Fluor 488, A-21206; Donkey Anti-Mouse IgG (H+L) Alexa Fluor 555, A-31570; Invitrogen
Validation	All antibodies used in this study validated by the manufactures or by the Namekawa laboratory.<br>The specificity of the H2AK119ub antibody for IF was validated by Maezawa, S. et al. (2017) (http://genesdev.cshlp.org/content/31/16/1693) and for Cut&Run by manufacture (https://www.cellsignal.com/products/primary-antibodies/ubiquityl-histone-h2a-lys119-d27c4-xp-rabbit-mab/8240) .The specificity of the H3K27me3 and H3K4me3 antibodies for CUT&RUN were validated by manufacture (https://www.cellsignal.com/products/primary-antibodies/tri-methyl-histone-h3-lys27-c36b11-rabbit-mab/9733?

site-search-type=Products&N=4294956287&Ntt=h3k27me3&fromPage=plp). The other antibodies used for IF have been validated by Namekawa lab (https://doi.org/10.1016/j.cub.2019.11.064).

Animals and other organisms

Policy information about studies involving animals; ARRIVE guidelines recommended for reporting animal research

Laboratory animals	Mice were maintained on a 12:12 light:dark cycle in a temperature and humidity-controlled vivarium (22±2°C; 40–50% humidity) with free access to food and water in the pathogen-free animal care facility.<br>Generation conditionally deficient Rnf2 mice on a Ring1-/- background was performed as described previously. Briefly, PRC1cKO mice Ring1-/-; Ring1F/-; Ddx4-Cre were generated from Ring1-/-; Ring1F/F females crossed with Ring1-/-; Ring1F/+; Ddx4-Cre males and PRC1ctrl mice used in experiments were Ring1-/-; Rnf2F/+; Ddx4-Cre littermate females. Female mice at P1, P5, 1m, 2m and 4m were used for oocyte collection or histological analyses. Mice were maintained on a mixed genetic background of FVB and C57BL/6J. Generation of mutant Ring1 and Rnf2 floxed alleles were reported previously. Ddx4-Cre transgenic mice were purchased from the Jackson Laboratory. Stella-GFP transgenic mice were obtained from Dr. M. Azim Surani. Neonatal P1 female pups of Stella-GFP transgenic mice were used for collecting oocytes.
Wild animals	no wild animals were used in the study.
Field-collected samples	no field collected samples were used in the study.
Ethics oversight	Mice were maintained and used according to the guidelines of the Institutional Animal Care and Use Committee (IACUC: protocol no. IACUC2018-0040 and 21931) at Cincinnati Children's Hospital Medical Center and the University of California, Davis.

Note that full information on the approval of the study protocol must also be provided in the manuscript.

ChIP-seq

Data deposition

☒ Confirm that both raw and final processed data have been deposited in a public database such as GEO.

☒ Confirm that you have deposited or provided access to graph files (e.g. BED files) for the called peaks.

Data access links<br>*May remain private before publication.*	https://www.ncbi.nlm.nih.gov/geo/query/acc.cgi?acc=GSE184208
Files in database submission	GSE184208 Polycomb establishes oocyte epigenome to produce ovarian<br><br>GSM5580259 RNAseq_P1NGO_PRC1ctrl_rep1<br>GSM5580260 RNAseq_P1NGO_PRC1ctrl_rep2<br>GSM5580261 RNAseq_P1NGO_PRC1cKO_rep1<br>GSM5580262 RNAseq_P1NGO_PRC1cKO_rep2<br>GSM5580263 RNAseq_P5NGO_PRC1ctrl_rep1<br>GSM5580264 RNAseq_P5NGO_PRC1ctrl_rep2<br>GSM5580265 RNAseq_P5NGO_PRC1cKO_rep1<br>GSM5580266 RNAseq_P5NGO_PRC1cKO_rep2<br>GSM5580267 CUTRUN_H3K4me3_PRC1ctrl_rep1<br>GSM5580268 CUTRUN_H3K4me3_PRC1ctrl_rep2<br>GSM5580269 CUTRUN_H3K4me3_PRC1cKO_rep1<br>GSM5580270 CUTRUN_H3K4me3_PRC1cKO_rep2<br>GSM5580271 CUTRUN_H3K27me3_PRC1ctrl_rep1<br>GSM5580272 CUTRUN_H3K27me3_PRC1ctrl_rep2<br>GSM5580273 CUTRUN_H3K27me3_PRC1cKO_rep1<br>GSM5580274 CUTRUN_H3K27me3_PRC1cKO_rep2<br>GSM5580275 CUTRUN_H2AK119ub_WT_rep1<br>GSM5580276 CUTRUN_H2AK119ub_WT_rep2
Genome browser session<br>(e.g. UCSC)	not available

Methodology

Replicates	CUT&RUN were obtained from biological duplicates.
Sequencing depth	For CUT&RUN, on average 15 million PE100 reads per replicate were obtained.
Antibodies	CUT&RUN library generation and sequencing:<br>The antibodies used were rabbit anti-H2AK119ub1 (1/100; Cell Signaling Technology; 8240), rabbit anti-H3K4me3 (1/100; Cell Signaling Technology; 9751), and rabbit anti-H3K27me3 antibody (1/100; Cell Signaling Technology; 9733).
Peak calling parameters	SEACR (https://seacr.fredhutch.org/) was used for peak calling with default parameters.

Data quality	Quality control (adapters and low-quality reads trimming) was performed by Trim-galore (version 0.6.6).
Software	Trim-galore (version 0.6.6), Bowtie2 (version 2.4.2), Picard tools (version 2.23.8), deepTools (version 3.5.0), bedtools (version 2.29.2), SEACR (https://seacr.fredhutch.org/), ngs.plot (version 2.63), Bamscale package(version 0.0.5), Enricher website (https://maayanlab.cloud/Enrichr/), ggplot2 (3.3.6).
